# Effect of different cleaning procedures on water use and bacterial levels in weaner pig pens

**DOI:** 10.1371/journal.pone.0242495

**Published:** 2020-11-17

**Authors:** Shilpi Misra, Corina E. van Middelaar, Kieran Jordan, John Upton, Amy J. Quinn, Imke J. M. de Boer, Keelin O’Driscoll

**Affiliations:** 1 Pig Development Department, Animal and Grassland Research and Innovation Centre, Teagasc, Moorepark, Fermoy, Ireland; 2 Animal Production Systems Group, Wageningen University & Research, Wageningen, The Netherlands; 3 Teagasc Food Research Centre, Moorepark, Fermoy, Ireland; 4 Livestock Systems Department, Animal and Grassland Research Innovation Centre, Teagasc Moorepark, Fermoy, Ireland; University of California Riverside, UNITED STATES

## Abstract

Pork is one of the most globally eaten meats and the pig production chain contributes significantly to the water footprint of livestock production. However, very little knowledge is available about the on-farm factors that influence freshwater use in the pig production chain. An experiment was conducted to quantify the effect of three different washing treatments on freshwater use, bacterial levels [(total bacterial counts; TBC), *Enterobacteriaceae* and *Staphylococcus*] and cleaning time in washing of pens for weaning pigs. Three weaner rooms were selected with each room having 10 pens and a capacity to hold up to 14 pigs each. Pigs were weaned and kept in the pens for 7 weeks. Finally, the pens were cleaned before the next batch of pigs moved in. The washing treatments used were power washing and disinfection (WASH); presoaking followed by power washing and disinfection (SOAK), and presoaking followed by detergent, power washing and disinfection (SOAK + DETER). A water meter was used to collect water use data and swab samples were taken to determine the bacterial levels. The results showed that there was no overall effect of washing treatments on water use. However, there was an effect of treatment on the washing time (*p*<0.01) with SOAK and SOAK+DETER reducing the washing time per pen by 2.3 minutes (14%) and 4.2 minutes (27%) compared to WASH. Nonetheless, there was an effect of sampling time (before or after washing) (*p*<0.001) on the levels of TBC and *Staphylococcus*, but no effect was seen on *Enterobacteriaceae* levels. Thus, the washing treatments used in this study had no effect on the water use of the pork production chain. Although there was no difference in both water use and bacterial load, from a producer perspective, presoaking and detergent use can save time and labour costs, so this would be the preferred option.

## 1 Introduction

Depletion of freshwater resources is an important environmental issue, with the livestock sector being responsible for 33% of global water withdrawals [1]. Freshwater use in livestock production is often quantified using “water footprinting” [2], which can be defined as the volume of freshwater used per unit of product produced (usually m^3^/ton). It is divided into green (rain water evapotranspired during crop cultivation or embedded in crops), blue (irrigation water) and grey (virtual freshwater used to assimilate pollution [3]) water. Pork is one of the most globally eaten meat products and its consumption is projected to increase further [4]. Detailed knowledge of blue water use in pig production and the on-farm factors that influence it is missing from the literature; the weighted global average blue water footprint for pork is 459 m^3^/ton (cradle-to-farm gate), which is approx. 9% of the estimated water footprint including green and blue water, and the environmental consequences can be significant [2, 5]. Insight into reduction options, therefore, is essential [5].

Several studies concluded that in the pig production chain, on-farm activities are the second largest contributor to blue water after feed production [6–8]. In US pork production systems, on-farm water use consisted of 87% for drinking, 5% for washing, 6% for cooling and 2% for other uses [9]. So far, there is very little literature available on water use for washing on pig farms; a conference article by Hurnik [10] compared different washing techniques and concluded that hot water reduced washing times by an average of 22%, presoaking by 50% and cleaning agents (soap) by an average of 12%, and that disinfectants reduced bacterial load. However, there was no information provided on the volume of water used. The cleaning process, as described by Sinner’s circle, is a combination of four factors; temperature, mechanical action, cleaning time and chemical action. These factors determine the efficiency of cleaning along with the properties of the surface to be cleaned [11]. Thus, changing the washing protocol, furthermore, can impact the hygiene on pig farms. In intensive pig production systems in Europe, strict biosecurity protocols and good hygiene are essential to reduce the risk of disease outbreaks, which can cause significant economic losses and have an important impact on animal welfare [12].

Pigs in intensive systems are transferred to different accommodation types during the production cycle and the washing of pens between batches of pigs is important, particularly for the younger pigs, including newly weaned pigs, which are more vulnerable to infectious diseases [13]. However, there is not much literature available about the efficacy of different cleaning and disinfection treatments in reducing bacterial load for younger age categories of pigs; most cleaning and disinfection studies focus on efficacy of washing procedures in finisher sections of the pig facilities, or lairage pens in slaughterhouses. Mannion [14] studied the efficacy of washing and disinfecting finisher units of different pig farms in reducing or eliminating levels of *Enterobacteriaceae*, and found a significant reduction in levels of *Enterobacteriaceae* on the pen floors, but no significant reduction was seen for feeder/drinker surfaces. Indeed, there was a significant increase in *Enterobacteriaceae* levels detected in the feeders following washing and disinfection. A study by Arguello [15] evaluated the effectiveness of cleaning and disinfection treatments against *Salmonella* on finishing farms, transport and lairage, and found that *Salmonella* persisted on 22.2% of the finisher farms, even after washing and disinfection procedures. Moreover, neither of these studies included measurement of water use. For newly weaned pigs, it would be useful to determine the levels of *Enterobacteriaceae* in the pens after cleaning because it is an important cause of a wide range of diseases especially post weaning diarrhoea, and this can cause significant economic losses [13]. *Staphylococcus* spp. (species) should also be determined as they are responsible for exudative epidermidis, abscesses and other conditions [16]. Therefore, thorough cleaning and disinfection of facilities are essential, and further knowledge on the effect of cleaning and disinfection on freshwater use and bacterial load is required.

Thus, the aim of this study was to quantify the effect of three different washing treatments on water use, bacterial levels and cleaning time in washing of weaner pig pens.

## 2 Material and methods

### 2.1 Experimental facilities

The study was conducted in the Teagasc Moorepark Pig Research Facility, which is a farrow to finish experimental pig unit with a 200 sow herd. Three rooms appropriate for housing newly weaned pigs were used for the experiment. Each room had 10 pens (2.4 m × 2.6 m) with a capacity to hold up to 14 pigs each. All the pens had fully slatted plastic floors with a single space wet-dry feeder in each pen, as well as a separate nipple drinker. The room temperature was maintained between 22–28°C. All the pigs received Moorepark standard weaner diet (15% barley, 23% wheat, 20% soya and 33% maize). On this farm, pigs remain in the weaner stage for 7 weeks, and then are moved to the finisher house. The pens are then cleaned before the next batch of pigs move in. The experiment was carried out over 3 replicates, every 7 weeks from April to August 2019.

### 2.2 Washing and disinfection treatments

Three washing treatments were evaluated: 1) power washing and disinfection (WASH), 2) presoaking followed by power washing and disinfection (SOAK), and 3) presoaking followed by detergent, power washing and disinfection (SOAK+DETER). Within each replicate one of the three treatments was randomly assigned to each experimental room, so that by the end of the experiment each room had each treatment applied once. No mechanical pre-treatment was done before the washing treatments and all the washing procedures were done by the same person. All the pens were washed from top to bottom i.e. first the feeders and walls were washed then the floor. It was only possible to apply treatments at room level, as the sprinklers covered the entire area, and thus it was not possible to separate the treatments within a room. Within each room and replicate, three pens were randomly selected for use in the experiment. Thus, by the end of all three replicates, 9 out of the 10 pens within each room had been used, as no pen was ever used twice.

For WASH, the treatment consisted of cold water (10–15°C, pH-7.53, conductivity-896μs/cm) washing using the power hose. For SOAK and SOAK+DETER treatments, the cold water (supply water at 10–15°C) sprinklers in each room were turned on for approximately 1h 40 minutes, and the detergent was applied for approximately 1h 35 minutes. At that point in the SOAK treatment the pens were washed as before, but for the SOAK+DETER treatment, detergent was applied with the cold water. The detergent used was Kenosan (CID lines, Belgium) used at 0.5% recommended dilution rate. All pens were disinfected after washing using Hyperox (Virkon, LANXESS Deutschland GmbH, Germany), a colourless aqueous formulation of peracetic acid, hydrogen peroxide, acetic acid and surfactant used at 1% recommended dilution rate. After the power washing, rooms were left to dry for 24h before applying disinfectant and after application of disinfectant, the rooms were left to dry for 48h.

### 2.3 Water data collection

A calibrated water meter (Shanxi Solid Industrial Co., Ltd., China) was installed on the power washing line (3000 psi and 14 L/min) and the volume of water used and the time taken to power wash each of the experimental pens was recorded. The time for which sprinklers were operating for the presoaking was also recorded. In total, there were 9 water recordings per room and per cleaning treatment.

### 2.4 Swab sample collection

To determine the efficacy of the different cleaning treatments, swab samples were collected from the floor, feeder and wall of each experimental pen. Sponge swabs (1.5 x 3-inch biocide-free cellulose sponge, pre-hydrated with a Neutralizing Buffer diluent; 3M Health Care, Minneapolis, USA) were used. In each pen, after it was emptied of pigs and before the implementation of the cleaning treatment, two floor swabs, one wall and one feeder swab, were collected. The feeder was made of metallic material and the swabs were collected from inside the feeders. The material of the walls and floor were plastic, floor samples were collected from the middle and side of the pen to get a representative sample. The floor of the pen was plastic slats so the surface area swabbed was 23cm x 23 cm (approx.). The swabs used for wall sampling covered an area of 30cm x 30cm and the swab used for the feeder covered 10cm x 10cm.

After washing and drying, swab samples were collected from the three rooms in the same way as before washing. Controls and blanks were used for the microbiological methods. All the swab samples were collected aseptically between 1600-1700h and stored overnight at 4°C and processed within 24h.

### 2.5 Microbiological analysis

Each swab was suspended in 90ml Maximum Recovery Diluent (MRD; Oxoid, Basingstoke, UK), homogenized in a Seward stomacher 400 (West Sussex, UK) for 1min and a ten-fold dilution series was performed in MRD. Relevant dilutions were plated in duplicate as follows (1) plated on 3M Petrifilm Aerobic count plates (3M Health Care, Minneapolis, USA), incubated at 30°C for 48h for total bacterial count (TBC); (2) pour-plated with Violet Red Bile Glucose (VRBG; Oxoid) agar which was overlaid and incubated at 37°C for 24h for *Enterobacteriaceae*; (3) spread plated on Baird Parker agar (Merck, Damstadt, Germany) mixed with Egg Yolk Tellurite Emulsion (VWR, Dublin, Ireland), incubated at 37°C for 48 h for *Staphylococcus*. For *Enterobacteriaceace*, the limit of detection was 10 CFU/cm^2^ for floor and wall swabs, and 100 CFU/cm^2^for feeder swabs. For *Staphylococci*, the limit of detection was 100 CFU/cm^2^ for floor and wall swabs, and 1000 CFU/cm^2^ for feeder swabs. For TBC, the limit of detection was 10 CFU/cm^2^ for floor and wall swabs, and 100 CFU/cm^2^ for feeder swabs.

### 2.6 Statistical analysis

All data were analysed using SAS version 9.4 (SAS Institute Inc., Cary, NC, USA). Prior to analysis the data were examined to visualize the distribution (PROC UNIVARIATE). The water use data were analyzed using linear mixed models (PROC MIXED). The model included washing treatment, replicate and room, as fixed effects, the number of pigs in the pen (11.6 ± 1.4) as a covariate, and pen nested within room as a random effect.

The TBC, *Enterobacteriaceae*, and *Staphylococcus* data were log transformed and analyzed using a linear mixed model (PROC MIXED). Our model included treatment, timing (i.e. before or after the treatment was applied), swab location, replicate and relevant interactions (treatment*timing and timing*location) as fixed effects, the number of pigs in the pen as a covariate, and the pen nested within room as a random effect. Time of sampling (i.e. before or after cleaning) was included as a repeated effect. The water use data and bacterial count data has been added as S1 and S2 Datasets. The SAS codes are given in S1 File. Residuals were checked graphically to ensure that the assumptions of the analyses were met. For all analyses, statistical significance was established at α ≤ 0.05. In all cases, the Tukey-Kramer least squares means adjustment for multiple comparisons was used to separate the treatment means.

## 3 Results

### 3.1 Water use

The effect of treatment on the time taken for washing each pen and the water used for washing are presented in (Table 1). There were no overall effects of treatment, or pair-wise differences, with regard to any measure of water use. There was an overall effect of treatment on the time taken to wash a pen (*p* < 0.01), with differences between all pairs of treatments. The WASH treatment took longer than both SOAK (*p* < 0.01) and SOAK+DETER (*p* < 0.001), whereas SOAK took longer than SOAK+DETER (*p* < 0.05). Thus, both presoaking and use of detergent reduced the time taken for pen washing. Detailed water use data is mentioned in S1 Dataset.

**Table 1:** Effect of cleaning and disinfection treatments on time taken for washing and the total water used (LSmeans±SE).

	WASH	SOAK	SOAK+DETER	*p*-value
Time/pen (min)	15.7 ± 0.5^a^	13.4 ± 0.5^b^	11.5 ± 0.5^c^	0.001
Water use/wash/pen (L)^1^	196.9 ± 18.2	191.1 ±17.7	179.1 ± 27.1	ns
Water use/pig (L)	16.5 ± 1.6	19.2± 1.5	18.3 ± 2.3	ns
Water use/pigspace/year (L)^3^^,^^4^	99.0 ± 9.5	114.2 ± 9.2	108.6 ± 14.0	ns
Total water use/pen (L)^2^	196.4 ± 18.8	226.6 ± 18.2	215.4 ± 27.9	ns

Treatments: WASH: cold water power washing, SOAK: presoaking using sprinkler followed by power washing, SOAK+DETER: presoaking using sprinkler followed by detergent application then power washing.

^1^ Water use per wash is the water used while power-hosing the pens.

^2^ Total water use/pen includes both water use per wash, and the volume of water used through the sprinklers.

^3^ Pigspace—0.42m^2^ per pig (represents the average floor space used by each pig/pen).

^4^ Values multiplied by 6 washes/year.

a,b,c Values within a row with different superscripts differ significantly at *p*<0.05.

ns–not significant.

### 3.2 Effect of treatment on bacterial counts

**Fig 1:**
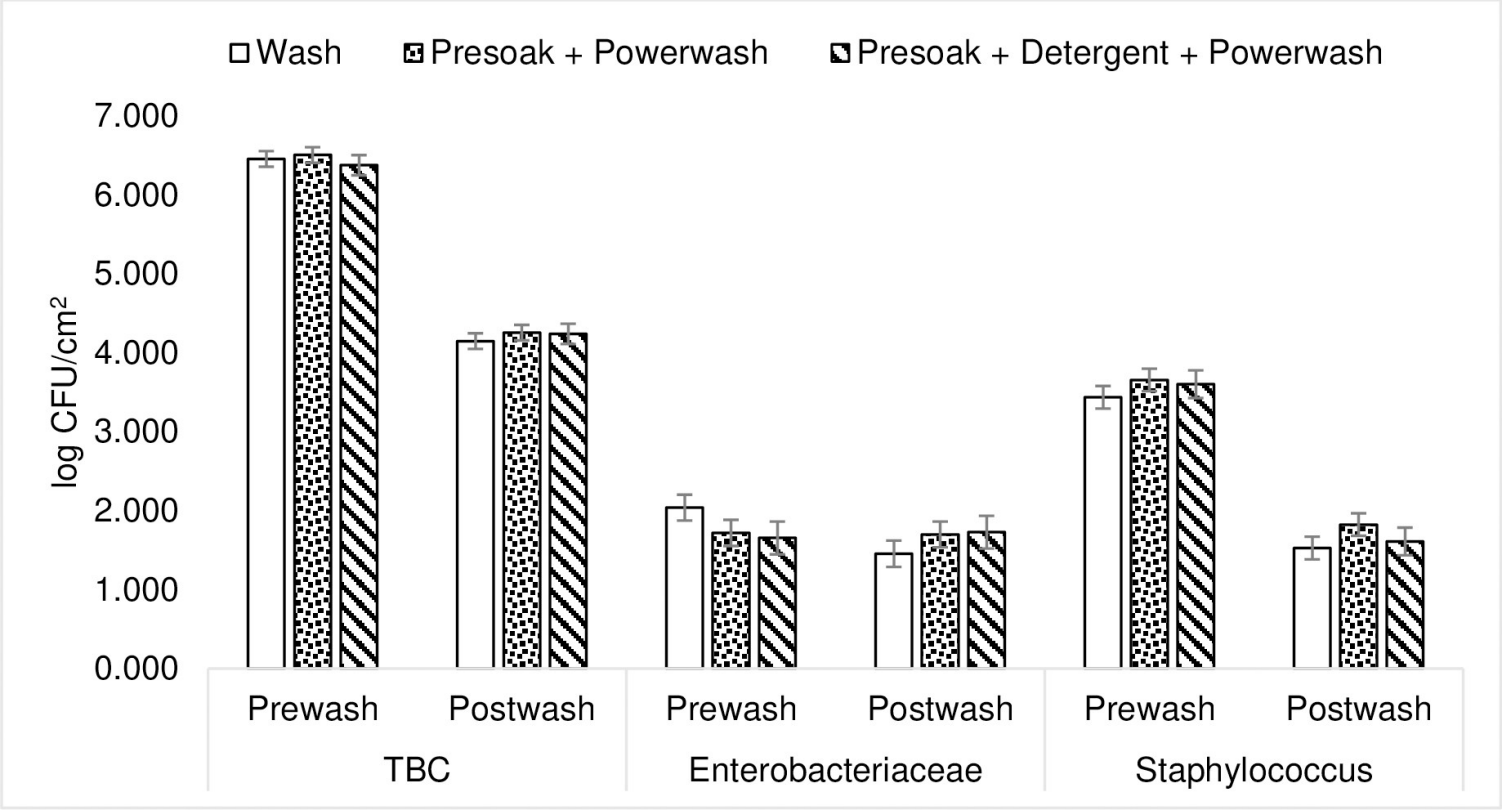
Effect of the different cleaning treatments on TBC (total bacterial count), *Enterobacteriaceae* counts and *Staphylococcus* counts in empty weaner pens before and after the treatments were applied (LSmeans±SE). There was no effect of treatment or interaction between treatment and sampling time for any measure. Treatments: WASH: cold water power washing, SOAK: presoaking using sprinkler followed by power washing, SOAK+DETER: presoaking using sprinkler followed by detergent application then power washing.

None of the treatments, nor the interaction between treatment and time (before or after washing), had any effect on any of the bacterial count measurements (Fig 1). Overall, the time of sampling (before or after wash) had an effect on both TBC and *Staphylococcus* counts (*p* < 0.001 for both), and within each treatment there was also an effect of time (before or after washing) (*p* < 0.001). However, there was no overall effect of time, or effect of time within treatment, on *Enterobacteriaceae* counts. Detailed bacterial count data is mentioned in S2 Dataset.

### 3.3 Effect of sampling location on bacterial counts

**Fig 2:**
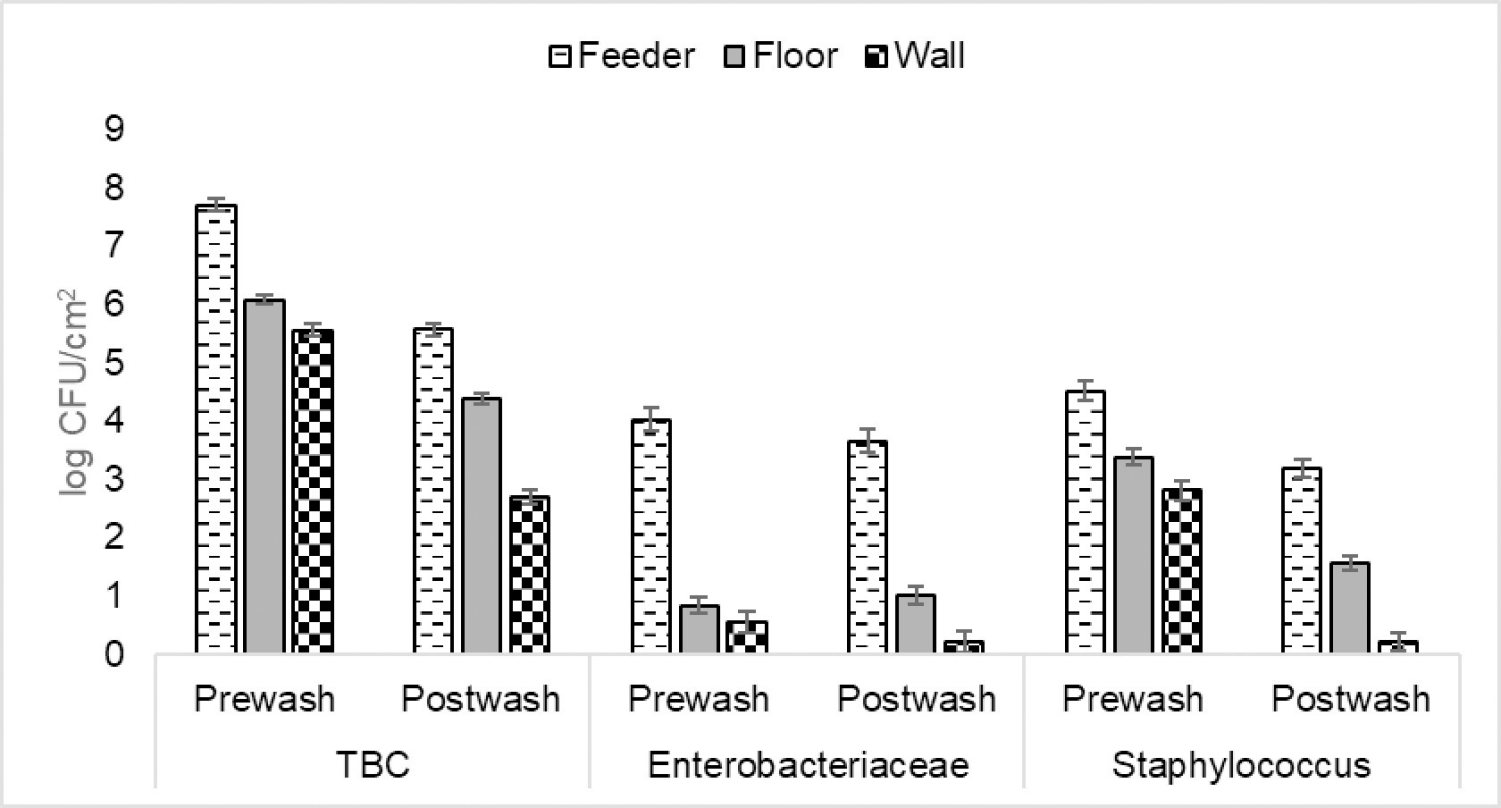
Effect of the different cleaning treatments on TBC (Total bacterial counts), *Enterobacteriaceae* counts and *Staphylococcus* counts in various locations in empty weaner pens before and after cleaning treatments were applied (LSmeans±SE). There was no effect of treatment or interaction between treatment and sampling time for any measure. Treatments: WASH: cold water power washing, SOAK: presoaking using sprinkler followed by power washing, SOAK+DETER: presoaking using sprinkler followed by detergent application then power washing.

The effect of sampling location and sampling time (before or after washing) on the bacterial counts is presented in (Table 2). After washing, there was a difference between counts at all locations (*p* < 0.001), indicating that washing of the walls had more of an effect in reducing bacterial load than washing of floors, regardless of the washing routine used. Again, for both TBC and *Staphylococcus*, the bacterial count was lower after washing than prior to washing, regardless of the location in the pen (*p* < 0.001 for all comparisons). However, the pattern for *Enterobacteriaceae* was different. There was no difference in *Enterobacteriaceae* count in any of the three locations before and after washing. Fig 2 shows the results of the bacterial counts based on the location of sampling before and after the cleaning treatments were applied.

**Table 2:** Significance of the effect of sampling location and timing (before or after washing) on the bacterial counts of weaner pig pens.

	Total bacterial count	*Enterobacteriaceae*	*Staphylococcus*
Location of sampling	P<0.001	P<0.001	P<0.001
Timing	P<0.001	ns	P<0.001
Location*Timing	P<0.001	ns	P<0.001

ns–not significant.

## 4 Discussion

In this study three washing treatments for weaner pig pens, with regard to the amount of water used, time taken for washing, and to their ability to reduce levels of TBC, *Enterobacteriaceae* and *Staphylococcus* were evaluated. Overall, it was shown that all three strategies were equally effective with regard to reducing bacterial load, and that they used the same volume of water. However, pre-soaking either alone or with a detergent significantly reduced the amount of time needed to clean the pens, and as such this strategy may have labor saving advantages for pig producers.

There is very little literature available about water use for washing in the pig industry, most of the research being focused on drinking water usage and water wastage for finishing houses. However, there are some studies that mention different cleaning methods and their effect on water use and cleaning efficiency. For example, Froese and Small [17] surveyed nine pig facilities in Manitoba and Saskatchewan, Canada, and found that the average daily washing water usage per pig for the nursery stage was 0.38 (L/day). A study done by a Swine Technical Group [18], reported that washing water usage for nursery stage was 10 L per piglet, 12 L/pigspace/wash, and that the time taken to wash a nursery pen was 1 minute per pigspace, which are lower than the values obtained in this study. They concluded that procedures such as use of detergent did not have a significant impact on wash timing, which is contradictory to the results of this study, where presoaking and use of detergent decreased washing time by 27% and presoaking decreased washing time by 14%, compared to only power-washing. The reason why there was no difference in time taken for washing even after use of detergent could be due to the lower concentration of the detergent used or less time given for presoaking. Moreover, washing treatments had no impact on the water use, which is similar to the results of this study.

In the current study even though the pre-soaking treatments (with or without detergent) used more water numerically, this was not detected as statistically significant. This could be a limitation of our study design. Based on the differences found, a larger scale study with 76 pens per treatment would be required to detect the differences in water use as statistically significant. The inclusion of sprinkler water in total water use, increased the water use for the SOAK and SOAK+DETER treatments. Thus, reducing the time for which sprinklers were running could reduce the water use while reducing washing time, but this needs to be studied further. However, when it came to water used purely for power-washing, the overall water use in these pens was lower than the WASH treatment, and the time it took to wash the pens was lower. Thus, when it comes to the reasons why producers may select various techniques, the benefits of reduced labor and time spent washing might outweigh any benefits of reduced water use.

The study by Hurnik, compared the effect of hot water to cold water washing, presoaking the pens, and the use of soap for washing pig pens in a finishing house in Canada [10]. In that study, it was found that when compared to only cold water pressure washing, use of hot water, along with presoaking and soap reduced the washing time by 31.2 minute or 45.9% per pen. Presoaking reduced the wash time by 26.6 minutes or 39.1% per pen and the use of soap with presoaking reduced the wash time by 31.7 minutes or 46.5% per pen [10]. In the current study, presoaking reduced the washing time by approximately 2.3 minutes or 14% per pen and use of detergent with presoaking reduced the washing time by approximately 4.2 minutes or 27% per pen. The time reduction was probably because presoaking helps to loosen the manure making it easier to clean. Presoaking also helps to break the biofilm and remove waxy residues which water alone cannot do, thus reducing the power washing time.

The efficacy of washing and disinfection strategies on bacterial reduction on pig farms has not been studied to a great extent and most of the studies focus on *Salmonella* prevalence in finishing houses or lairage pens. A study by Mannion [14] reported the efficacy of cleaning and disinfection in reducing or eliminating the levels of *Enterobacteriaceae* in finisher units on Irish pig farms. All the farms studied used cold high pressure washing with or without disinfectant. As in the current study, swab samples were collected before and after cleaning from the pen floors and feeder/drinkers. Although *Enterobacteriaceae* levels decreased moderately on the floors after cleaning, significant residual contamination remained on the surface of the feeder/drinker. Moreover, farms that washed without disinfection had little or no reduction in *Enterobacteriaceae* levels and a common trend among all farms was an increase in *Enterobacteriaceae* levels following cleaning and disinfection. These results are similar to those found in the current study. Cleaning and disinfection procedures were generally effective for TBC and *Staphylococcus* on pen floors and walls but contamination of feeder/drinkers is still of concern, probably due to difficulty in accessing all the crevices of feeders/drinkers. Indeed, the levels of *Enterobacteriaceae* did not decline from pre to post washing and disinfection. This could be due to the resistance of *Enterobacteriaceae* to the disinfectant used, or to the concentration of disinfectant being too low, or to the growth phase (log, lag or stationary phase) of the bacteria, which can influence bacterial reduction [19]. Cherci and Gu [19] used chlorine based disinfection although, Gosling recommends aldehyde-based disinfectants to be most effective in reducing bacterial counts [20].

Additionally, a recent study by Heinemann [21] about hygiene in pig fattening pens showed that even after the cleaning and disinfection procedures, the TVC was higher in drinkers/feeding areas compared to floors and walls. In general, it appeared that the feeders were the primary harborage site for *Enterobacteriaceae* which could be due to feed residues left in the feeders post cleaning, because of difficulty in cleaning the crevices of feeders. Other reasons as mentioned in the study by Mannion [14] include water splashing when pen floors are washed or generation of aerosol droplets due to high water pressure, thus reducing water pressure was recommended by them. Another factor as mentioned in a study by Hancox [22] is use of detergent in the cleaning regime. They concluded that detergents have their own bactericidal properties thus, soaking with detergent significantly reduced the total aerobic count and *Enterobacteriaceae*, depending on the target surface material.

Similar studies about the effectiveness of cleaning and disinfection procedures on farms have been done on other animal species. Martelli [23] studied the effect of cleaning and disinfection on *Salmonella* in duck farms and found detergent and formaldehyde are very effective against *Salmonella*. However, they also observed residual contamination on feeders/ drinkers after the cleaning procedures. Luyckx [24] evaluated the cleaning and disinfection procedures in broiler houses and found overnight soaking to be effective in reducing the total aerobic counts. The drain holes and the floor cracks were still infected with *E*. *coli* even after disinfection. Washing and disinfection procedures might help to remove the residual matter and bacteria from the pens but in most cases residual contamination remains in the inaccessible areas, which is a reason for concern. Moreover, water use in cleaning is still an area which needs further research. Use of detergent for cleaning might be of potential environmental concern if slurry containing detergent enters sewers or public waters. However, studying the environmental impact of detergents was not a part of this study.

## 5 Conclusions

The three washing treatments used in this study had no significant effect on water use but there was a significant difference in washing time. The cleaning treatments reduced the levels of *Staphyloccocus* and TBC from pre to post wash, even though no significant difference between the treatments was observed. On the other hand, the levels of *Enterobacteriaceae* did not decline post washing. Since there was no difference in both water use and bacterial load, power-washing without pre-soaking or detergent seems to be the simplest method, and thus perhaps the preferred option. However, if a view from the producer perspective is taken, presoaking and detergent use saves time and labour cost, so this would be the preferred option.

The information gathered in this study is useful for future research. Future research in this area should test different concentrations of detergent and disinfectant, a large scale study with more pens might show different results. However, increasing the concentrations of disinfectant and detergent might have some environmental consequences which need to be studied for optimizing the cleaning protocols.

## Supporting information

S1 Dataset This contains the water use data. (XLSX)

S2 Dataset This contains the bacterial count data. (XLSX)

S1 File This contains the SAS codes. (DOCX)
