# Supplementary material for: Effect of different cleaning procedures on water use and bacterial levels in weaner pig pens
Source: PLoS One. 2020 Nov 17;15(11):e0242495. doi: 10.1371/journal.pone.0242495 (PMC7671538; doi:10.1371/journal.pone.0242495)
Supplement: S1 File — (DOCX) [file pone.0242495.s003.docx]

**Effect of different cleaning procedures on water use and bacterial levels in weaner pig pens**

Shilpi Misra^1,2^, Corina E. van Middelaar^2^, Kieran Jordan^3^, John Upton^4^, Amy J. Quinn^1^, Imke J.M. de Boer^2^, Keelin O’Driscoll^1^

*^1^ Pig Development Department, Animal and Grassland Research and Innovation Centre, Teagasc, Moorepark, Co. Cork, P61 C996, Ireland*

*^2^Animal Production Systems Group, Wageningen University & Research, P.O. Box 338, 6700, AH, Wageningen, the Netherlands*

^3^Teagasc Food Research Centre, Moorepark, Fermoy, Co. Cork, P61 C996, Ireland

*^4^Animal and Grassland Research Innovation Centre, Teagasc Moorepark, Co. Cork, P61 C996, Ireland*

**SAS Mixed procedure Codes**

1. **Time taken for washing**

**proc** **mixed** data=Water;

class Replicate Room Treatment pen Time;

model Time = Treatment Replicate room pigs/ ddfm=kr residual;

random pen(Room)/solution;

LSmeans Treatment / pdiff = all adjust = tukey;

**run**;

1. **Total water use/pen**

**proc** **mixed** data=Water;

class Replicate Room Treatment pen Time;

model Water = Treatment Replicate room pigs/ ddfm=kr residual;

random pen(Room)/solution ;

LSmeans Treatment / pdiff = all adjust = tukey;

**run**;

1. **Wateruse/wash/pen**

**proc** **mixed** data=Water;

class Replicate Room Treatment pen Time;

model Powerwash = Treatment Replicate room pigs/ ddfm=kr residual;

random pen(Room)/solution ;

LSmeans Treatment / pdiff = all adjust = tukey;

**run**;

1. **Water use/pig (L)**

**proc** **mixed** data=Water;

class Replicate Room Treatment pen Time;

model Waterpig = Treatment Replicate room pigs/ ddfm=kr residual;

random pen(Room)/solution ;

LSmeans Treatment / pdiff = all adjust = tukey;

**run**;

1. **Water use/pigspace/year# (L)**

**proc** **mixed** data=Water;

class Replicate Room Treatment pen Time;

model Wateryear = Treatment Replicate room pigs/ ddfm=kr residual;

random pen(Room)/solution ;

LSmeans Treatment / pdiff = all adjust = tukey;

**run**;

1. **Total bacterial count (TBC)**

**proc** **mixed** data=avgbact;

class rep room treatment pen timing location2 sampleno;

Model tbc = treatment|timing timing|location2 rep pigs/ ddfm=kr residual ;

repeated timing / subject=location2(rep sampleno pen room) type = ar(**1**);

random pen(room);

LSmeans treatment|timing timing|location2 / pdiff = all adjust = tukey ;

ods output LSmeans =means Diffs =Pdiffs;

title 'tbc';

**run**;

1. **Staphylococcus**

**proc** **mixed** data=avgbact;

class rep room treatment pen timing location2 sampleno;

Model staph = treatment|timing timing|location2 rep pigs/ ddfm=kr residual ; /*no 3 way interaction*/

repeated timing / subject=location2(rep sampleno pen room) type = ar(**1**);

random pen(room);

LSmeans treatment|timing timing|location2 / pdiff = all adjust = tukey ;

ods output LSmeans =means Diffs =Pdiffs;

title 'Staph';

**run**;

1. **Enterobacteriaceae**

**proc** **mixed** data=avgbact;

class rep room treatment pen timing location2 sampleno;

Model entero = treatment|timing timing|location2 rep pigs/ ddfm=kr residual ; /*no 3 way interaction*/

repeated timing / subject=location2(rep sampleno pen room) type = ar(**1**);

random pen(room);

LSmeans treatment|timing timing|location2 / pdiff = all adjust = tukey ;

ods output LSmeans =means Diffs =Pdiffs;

title 'entero';

**run**;
